# Risk factors for recurrent cases of early-stage uterine sarcoma after complete surgical resection

**DOI:** 10.1186/s12885-026-15618-x

**Published:** 2026-01-26

**Authors:** Yukari Nagao, Akira Yokoi, Kosuke Yoshida, Masato Yoshihara, Satoshi Tamauchi, Nobuhisa Yoshikawa, Kaoru Niimi, Hiroaki Kajiyama

**Affiliations:** 1 Department of Obstetrics and Gynecology, Nagoya University Graduate School of Medicine, 65 Tsurumai-cho, Showa-ku, Nagoya, Aichi 466-8550 Japan; 2 Institute for Advanced Research, Nagoya University, Furo-cho, Chikusa-ku, Nagoya, Aichi 464-8603 Japan

**Keywords:** Uterine sarcoma, Uterine leiomyosarcoma, Low-grade endometrial stromal sarcoma, Recurrence, Prognostic factor

## Abstract

**Background:**

Uterine sarcoma has an inferior prognosis and high recurrence rate among gynecological malignancies, even in early-stage cases with complete resection. However, the risk factors for recurrence remain poorly understood. This study aimed to identify risk factors associated with recurrence in early-stage uterine sarcoma.

**Methods:**

Among 97 patients with uterine sarcoma treated at our institution between January 2007 and June 2023, we retrospectively investigated 55 patients of the following five histological types: uterine leiomyosarcoma (ULMS), low- or high-grade endometrial stromal sarcoma (LG-ESS or HG-ESS), adenosarcoma, and smooth muscle tumor of uncertain malignant potential (STUMP). Risk factors were compared between the recurrence and non-recurrence groups using univariate analysis, and recurrence rates, time to recurrence, progression-free survival (PFS), and overall survival (OS) were examined.

**Results:**

The median age of 55 patients was 48 years, and the most common initial symptom was abdominal pain or abdominal mass awareness (29.4%), followed by abnormal bleeding in 25.5% of the patients. The median tumor size was 9.7 cm, and stage I cases were 64.8% of the total. Histological types were 28 ULMS, 13 LG-ESS, 8 STUMP, 5 HG-ESS, and one adenosarcoma. Among stage I cases, ULMS had a recurrence rate of 81.3% with a median time to recurrence of 12.4 months, while LG-ESS had a recurrence rate of 30% with a median time to recurrence of 41.1 months. A high mitotic count was significantly associated with recurrence in stage I ULMS (*p* = 0.044). Other surgical pathological findings, such as lymphovascular space invasion, MIB-1 positive rate, and necrosis, and surgical factors, such as myomectomy and ovarian preservation, showed no statistically significant differences but were higher in the recurrence cases. The 5-year PFS rates in stage I ULMS and LG-ESS groups were 31.3% and 75%, and the 5-year OS rates were 68.5% and 100%, respectively.

**Conclusions:**

In stage I ULMS, a high mitotic count was associated with an increased risk of recurrence after complete surgical resection.

## Background

Uterine sarcomas are rare, accounting for 4%–9% of all uterine malignancies [1]. Uterine sarcoma has a particularly poor prognosis among gynecological malignancies, and the standard treatment is not well established. The prognosis greatly depends on the histological type and stage of disease. Indeed, the 5-year overall survival (OS) rate for low-grade endometrial stromal sarcoma (LG-ESS) is relatively good at over 90%, while the 5-year OS rate for high-grade endometrial stromal sarcoma (HG-ESS) is poor at 32.6% [2]. Uterine leiomyosarcoma (ULMS) has a poor prognosis even in early stages, with 5-year OS rates of 51%–55% for stage I and 25%–32% for stage II [3, 4]. Due to its aggressive clinical behavior, even in early-stage diseases that can be completely resected by surgery, especially for ULMS, the recurrence rate is high [5, 6]. Nevertheless, according to the National Comprehensive Cancer Network (NCCN) guidelines (Version 3.2025 Uterine Neoplasms), postoperative adjuvant therapy is not recommended for all stage I completely resected uterine sarcomas [7]. However, even in early-stage cases, some studies have reported that postoperative chemotherapy in ULMS increases the 5-year OS rate, while postoperative hormone treatment in LG-ESS suppresses the recurrence rate [8, 9].

In early-stage uterine sarcomas, complete surgical resection of the tumor by total hysterectomy and bilateral salpingo-oophorectomy (BSO) is recommended if medically operable [7]. However, in selected patients who express a strong desire to preserve fertility or hormonal function, preservation of the uterus or ovaries may be considered [7, 10, 11]. Similarly, if uterine sarcoma is diagnosed after myomectomy or ovarian preservation, additional surgery to complete a total hysterectomy and BSO is recommended, but the patient may choose not to undergo additional surgery only in selected cases, as described above [7]. In the NCCN guidelines, the only clearly defined poor prognostic factors, including recurrence risk, are those related to ovarian preservation in estrogen-dependent histology and intraperitoneal morcellation [7, 12–14]. Therefore, in general, BSO is recommended for LG-ESS, adenosarcoma, and tumors expressing estrogen receptor (ER)/ progesterone receptor (PR), while intraperitoneal morcellation is contraindicated. However, other risks of recurrence, such as those associated with pathological findings, are not clearly discussed. Thus, the choice of primary surgery or additional surgery after uterine or ovarian preservation surgery is usually based on a desire to preserve fertility or hormonal function. The protocols for selecting surgery and adjuvant therapy according to the recurrence risk factors are currently undefined. A clear understanding of the recurrence risk factors in stage I uterine sarcoma after complete resection helps to consider primary surgery, postoperative adjuvant therapy, and additional surgery. Therefore, the aim of this study was to investigate the characteristics of uterine sarcoma and identify risk factors for recurrence, particularly in cases of complete resection of stage I uterine sarcoma.

## Methods

**Fig. 1:**
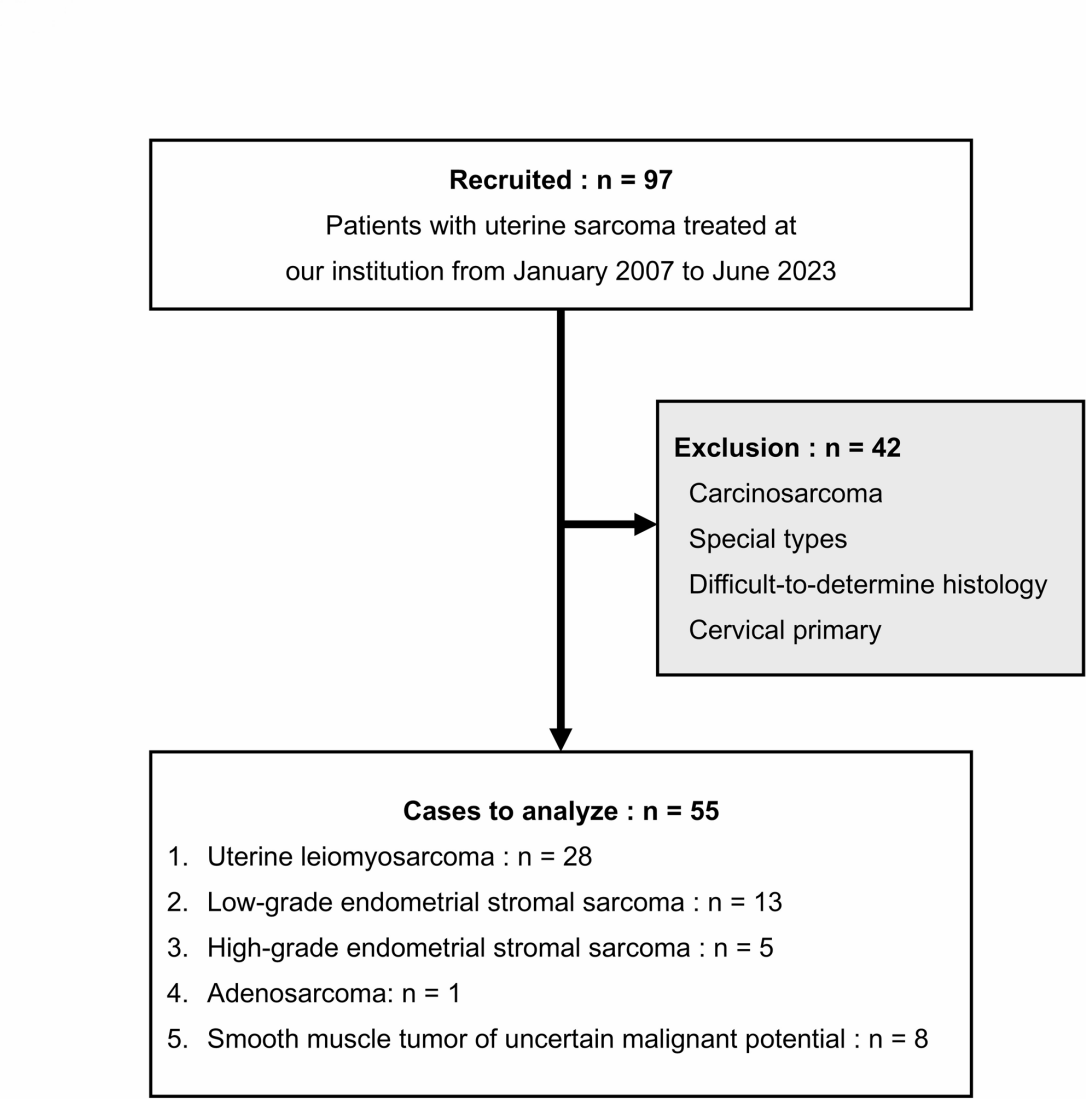
Flowchart of patient selection. Of the 97 patients with uterine sarcoma treated at our institution from January 2007 to June 2023, we excluded 42 patients with carcinosarcoma, special types, difficult-to-determine histology, and cervical primary, and included 55 patients with five histological types of uterine origin

Overall, 97 patients with uterine sarcoma who were treated at our institution from January 2007 to June 2023 were included in this study. Among these patients, 42 patients with carcinosarcoma, special types, difficult to determine histology, and cervical primary tumors were excluded from the study. A total of 55 patients with the following five histological types of uterine origin were included in the study: ULMS, LG-ESS, HG-ESS, adenosarcoma, and smooth muscle tumor of uncertain malignant potential (STUMP) (Fig. 1).

The study collected the following data by reviewing medical records: patient characteristics including age, history of delivery, menopausal status, and past medical history; presenting symptom and hematologic findings at diagnosis; tumor size, stage, primary treatment details, pathological findings, and ascites cytology results; adjuvant therapy; recurrence status, duration, site, and outcome. Some cases lacked available information for several parameters. When calculating values as n (%), the number of cases with available information was stated in the table footnote. For mitotic counts, we classified cases with ≥ 10 mitoses per 10 HPF as the high group and those with < 10 as the low group. However, some cases where the number was not specified were classified as high if the pathologist clearly diagnosed a high number of mitoses, and as low if the pathologist clearly diagnosed a low number.

This study was approved by the Ethics Committee of our institution (Approval No. 2023 − 0187). All methods were performed in accordance with relevant guidelines and regulations and in compliance with the Declaration of Helsinki. Furthermore, informed consent was obtained in the form of opt-out on the website. Further details are provided in the section “Ethics approval and consent to participate.”

Statistical analyses were performed using SPSS version 29 (IBM Corp., Armonk, NY). The Mann–Whitney U test and Fisher’s exact tests were used to compare the two groups. OS was defined as the time from the start of primary therapy to all-cause mortality. Progression free survival (PFS) was defined as the time from primary therapy to tumor progression, recurrence, and all-cause mortality. Kaplan–Meier curves were used to analyze OS and PFS, and the log-rank test was used to compare survival curves. Differences with *p* < 0.05 (two-tailed) were considered statistically significant.

## Results

The characteristics of 55 patients with uterine sarcoma included in this study are presented in Table 1. The median age of all patients was 48 years (range: 32–76), 66% of the patients had a history of delivery, and 32.1% were postmenopausal. Abdominal pain or abdominal mass awareness was the most common first presenting symptom (29.4%), followed by abnormal bleeding in 25.5% of the patients, while 17 of 51 patients (33.3%) were asymptomatic. The histological types of uterine sarcomas were ULMS in 28 patients (50.9%), LG-ESS in 13 (23.6%), STUMP in 8 (14.5%), HG-ESS in 5 (9.1%), and adenosarcoma in 1 (1.8%). The median tumor size was 9.7 cm (range: 2–25). Additionally, the FIGO 2008 stage was stage I in 35 patients (64.8%), stage II in 6 (11.1%), stage III in 3 (5.6%), and stage IV in 10 (18.5%). Among these, stage I cases of ULMS and LG-ESS were particularly studied in detail.

**Table 1:** Characteristics of all patients with uterine sarcoma

Variables	Total (*n* = 55)
Age, median (range)	48 (32–76)
Previous delivery, n (%) *	35 (66%)
Post menopause, n (%) *	17 (32.1%)
Presenting symptoms, n (%) ^†^	
No	17 (33.3%)
Yes	34 (66.7%)
Abdominal pain/ Abdominal mass awareness	15 (29.4%)
Abnormal bleeding	13 (25.5%)
Abnormal menstruation	10 (19.6%)
Urinary or defecation disorders	2 (3.9%)
Low back pain	2 (3.9%)
Fever	1 (2%)
Past medical history, n (%) ‡	
Hormonal therapy	3 (5.6%)
Breast cancer	0 (0%)
Ovarian cancer	0 (0%)
Histological type of sarcoma, n (%)	
ULMS	28 (50.9%)
LG-ESS	13 (23.6%)
HG-ESS	5 (9.1%)
Adenosarcoma	1 (1.8%)
STUMP	8 (14.5%)
Tumor size (cm), median (range)	9.7 (2–25)
Stage (FIGO 2008), n (%) ‡	
I A	7 (13%)
I B	26 (48.1%)
I (A or B unknown)	2 (3.7%)
IIB	6 (11.1%)
IIIA	1 (1.9%)
IIIB	1 (1.9%)
IIIC	1 (1.9%)
IVB	10 (18.5%)

*ULMS* Uterine leiomyosarcoma, *LG-ESS* Low grade endometrial stromal sarcoma, *HG-ESS* High grade endometrial stromal sarcoma, *STUMP* Smooth muscle tumor of uncertain malignant potential

* *n* = 53; ^†^ *n* = 51; ‡ *n* = 54; cases with information available

The characteristics of 16 patients with stage I ULMS are presented in Table 2, comparing non-recurrence (*n* = 3) and recurrence (*n* = 13) cases. The results revealed no significant differences in basic characteristics, including age, history of delivery, menopausal status, tumor size, hematologic findings at diagnosis, and surgical stage between the ULMS stage I non-recurrence and recurrence groups. All patients with stage I ULMS disease underwent surgery as the primary treatment, and eventually, total hysterectomy was performed in all patients. However, only in the recurrence group, myomectomy was performed prior to total hysterectomy in 1 patient (7.7%), and ovarian preservation, including both bilateral and unilateral, was also performed in 6 patients (46.2%). Although there was no significant difference between the non-recurrence and recurrence groups, there were no cases of either prior myomectomy or ovarian preservation in the non-recurrence group. In addition, regarding surgical pathological findings, when a mitotic count was classified as high or low group, the recurrence group had significantly higher mitotic counts (*p* = 0.044; Fisher’s exact tests). There was no significant difference in other surgical pathological findings between the two groups; however compared with the non-recurrence group, the recurrence group had a higher rate of positive lymphovascular space invasion (LVSI) (16.7% vs. 0%) and more necrosis (75% vs. 33.3%). The recurrence rate for stage I ULMS was 81.3%, with a median time to recurrence of 12.4 months (range: 0.4–75.5). The recurrence sites of the patients were the lung in 8 patients (61.5%), peritoneal seeding or intra-abdominal tumor in 7 (53.8%), bone in 5 (38.5%), liver or lymph node in 1 (7.7%), and others in 2 (15.4%).

**Table 2:** Characteristics of patients with stage I uterine leiomyosarcoma

Variables	Non-recurrence (*n* = 3)	Recurrence (*n* = 13)	*p* value
Age (years), median (range)	44 (39–49)	52 (33–76)	0.296
Previous delivery, n (%) ^§^	3 (100%)	8 (72.7%)	1
Post menopause, n (%) ^§^	0 (0%)	4 (36.4%)	0.505
Tumor size (cm), median (range)	12 (2.5–14)	9.7 (4.8–25)	0.937
Hematologic findings at diagnosis, median (range)			
WBC (/µl)	6,250 (5,300–7,200)	5,650 (4,300–11,600)	0.857
Neutrophil (/µl)	4,350 (3,700–5,000)	4,450 (2,000–8,700)	1
Hb (g/dl)	9.6 (7.2–12)	10.2 (7.6–12.5)	1
CRP (mg/dl)	0.18	1.21 (0.04–9.56)	1
LDH (U/l)	217.5 (150–285)	338 (174–1237)	0.333
CA125 (U/ml)	45.9	39.5 (4.4–114.4)	0.750
Surgical stage (FIGO 2008), n (%)			0.396
I A	1 (33.3%)	1 (7.7%)	
I B	2 (66.7%)	10 (76.9%)	
I (A or B unknown)	0 (0%)	2 (15.4%)	
Primary treatment, n (%)			
Total hysterectomy performed, n (%)	3 (100%)	13 (100%)	―
Myomectomy prior to total hysterectomy, n (%)	0 (0%)	1 (7.7%)	1
Ovarian preservation, n (%)	0 (0%)	6 (46.2%)	0.250
Positive ascites cytology, n (%)	0 (0%)	0 (0%)	―
Pathological findings			
LVSI, n (%) ^‖^	0 (0%)	2 (16.7%)	1
MIB-1 (%), median (range)	8 (3.5–12.5)	9.5 (0.5–25)	1
High mitotic count, n (%) ^‖^	0 (0%)	9 (75%)	**0.044**
Necrosis, n (%) ^‖^	1 (33.3%)	9 (75%)	0.242
Adjuvant therapy, n (%)	0 (0%)	2 (15.4%)	1
Time to recurrence (months), median (range)		12.4 (0.4–75.5)	―
Recurrence site, n (%)			―
Lung		8 (61.5%)	
Peritoneal seeding/intra-abdominal tumor		7 (53.8%)	
Bone		5 (38.5%)	
Liver		1 (7.7%)	
Lymph node		1 (7.7%)	
Others		2 (15.4%)	

*LVSI* Lymphovascular space invasion

In the recurrence group, information was available for ^§^ *n* = 11 and ^‖^ *n* = 12 cases

High mitotic count: defined as ≥ 10 mitoses per 10 HPF. Includes some cases with an unspecified number but diagnosed by pathologists as clearly high

Only the *p*-value indicating a significant increase in high mitotic count is shown in boldface

The characteristics of the 10 patients with stage I LG-ESS disease are presented in Table 3, comparing non-recurrence (*n* = 7) and recurrence (*n* = 3) cases. The results revealed no significant differences in basic characteristics between the non-recurrence and recurrence groups of stage I LG-ESS. All patients in stage I LG-ESS underwent surgery as primary treatment, and eventually all patients in the non-recurrence group underwent total hysterectomy, whereas 1 patient (33.3%) in the recurrence group completed only myomectomy. Three patients (42.9%) in the non-recurrence group and 2 (66.7%) in the recurrence group underwent myomectomy. Similarly, ovarian preservation was performed in 3 patients (42.9%) in the non-recurrence group and 2 (66.7%) in the recurrence group. Additionally, there was no significant difference in surgical pathological findings between the two groups, but compared to the non-recurrence group, the recurrence group had a higher rate of positive LVSI (33.3% vs. 0%), a higher MIB-1 positive rate (5.75% vs. 2.5%), and a higher rate of high mitotic count (33.3% vs. 0%). There were no positive ascites cytology cases in both groups, but one case in the recurrence group was suspicious-positive. The recurrence rate for stage I LG-ESS was 30%, with a median time to recurrence of 41.1 months (range: 6.0–75.5). The recurrence sites of three patients were each lung, peritoneal seeding, and the uterus.

**Table 3:** Characteristics of patients with stage I low grade endometrial stromal sarcoma

Variables	Non-recurrence (*n* = 7)	Recurrence (*n* = 3)	*p* value
Age (years), median (range)	44 (32–65)	36 (33–49)	0.667
Previous delivery, n (%)	5 (71.4%)	1 (33.3%)	0.500
Post menopause, n (%)	1 (14.3%)	0 (0%)	1
Tumor size (cm), median (range)	6.4 (2.3–10.7)	5.1 (2–6.5)	0.517
Surgical stage (FIGO 2008), n (%)			1
I A	3 (42.9%)	1 (33.3%)	
I B	4 (57.1%)	2 (66.7%)	
Biopsy before primary treatment, n (%)	1 (14.3%)	0 (0%)	1
Primary treatment, n (%)			
Total hysterectomy performed, n (%)	7 (100%)	2 (66.7%)	0.300
Myomectomy performed, n (%)	3 (42.9%)	2 (66.7%)	1
Ovarian preservation, n (%)	3 (42.9%)	2 (66.7%)	1
Positive ascites cytology, n (%)	0 (0%)	0 (0%)	―
Pathological findings			
LVSI, n (%)	0 (0%)	1 (33.3%)	0.300
MIB-1 (%), median (range)	2.5 (0.5–7.5)	5.75 (1.5–10)	0.429
High mitotic count, n (%)	0 (0%)	1 (33.3%)	0.300
Necrosis, n (%)	1 (14.3%)	1 (33.3%)	1
Adjuvant therapy, n (%)	0 (0%)	0 (0%)	―
Time to recurrence (months), median (range)		41.1 (6.0–75.5)	―
Recurrence site			―
Lung		1 (33.3%)	
Peritoneal seeding/intra-abdominal tumor		1 (33.3%)	
Uterus		1 (33.3%)	

*LVSI* Lymphovascular space invasion

Myomectomy performed: includes both prior myomectomy before hysterectomy and myomectomy-only cases

High mitotic count: defined as ≥ 10 mitoses per 10 HPF. Includes some cases with an unspecified number but diagnosed by pathologists as clearly high

**Fig. 2:**
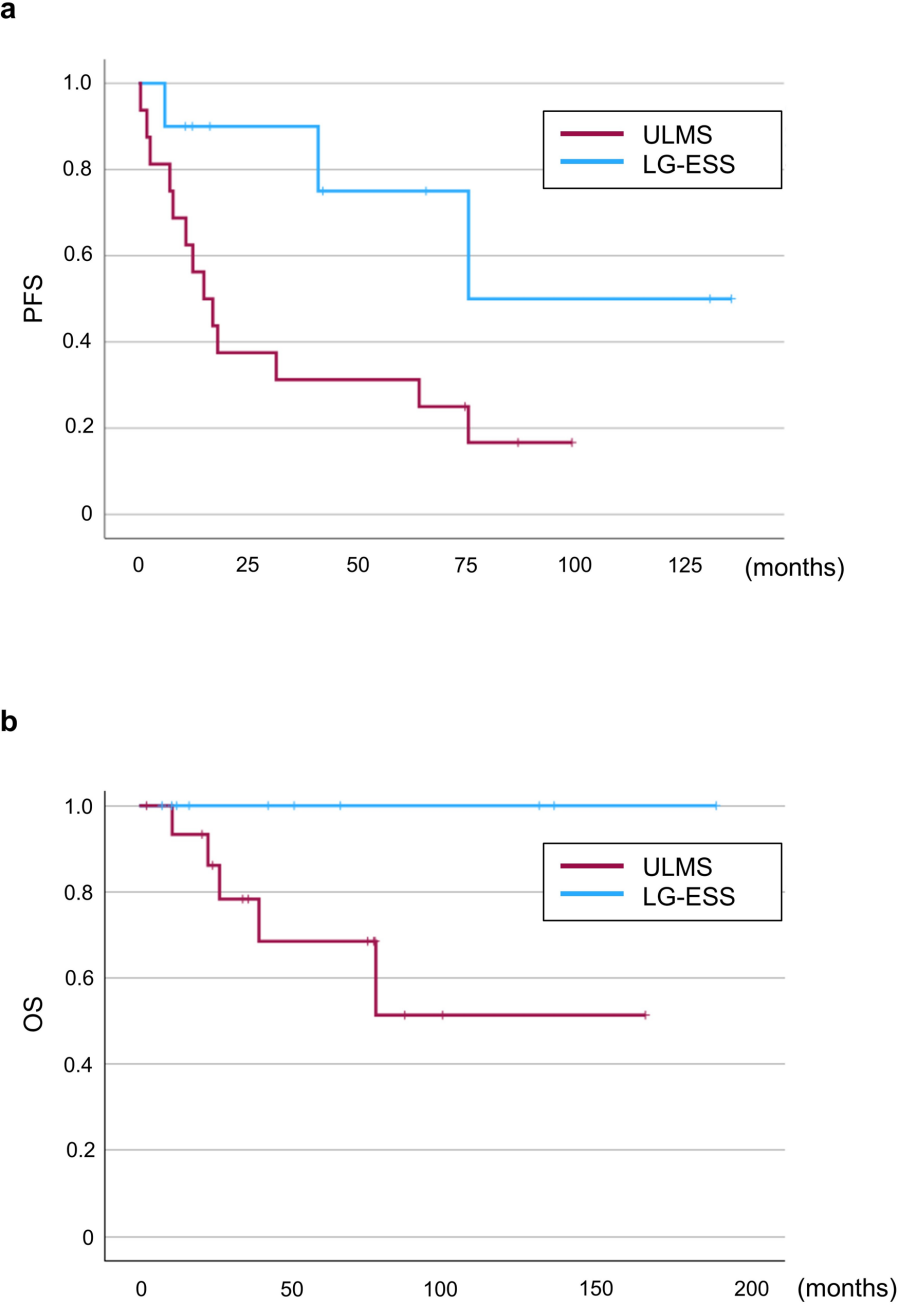
Prognosis of patients with stage I uterine leiomyosarcoma (ULMS) and low-grade endometrial stromal sarcoma (LG-ESS). Kaplan–Meier curves showing (**a**) progression-free survival (PFS) and (**b**) overall survival (OS) in patients with stage I ULMS and stage I LG-ESS

The 5-year PFS rates in stage I ULMS and stage I LG-ESS groups were 31.3% and 75%, respectively (Fig. 2a). The 5-year OS rates in stage I ULMS and stage I LG-ESS groups were 68.5% and 100%, respectively (Fig. 2b). Kaplan–Meier curves indicated a statistically significant difference between the two groups in terms of PFS (*p* = 0.033; log-rank test), but not in terms of OS (*p* = 0.082; log-rank test).

## Discussion

Among gynecological malignancies, uterine sarcoma is an aggressive disease with a poor prognosis [2–4]. Recurrence rates are high even in early-stage uterine sarcomas, especially ULMS [5, 6], but risk factors for recurrence after complete resection of early-stage uterine sarcomas are not clearly defined, and postoperative adjuvant therapy is not uniformly recommended for stage I complete resection [7]. Additionally, the decision on ovarian preservation is based on age and a strong desire for fertility preservation, rather than on the risk of recurrence, except for the risk factor of estrogen-dependent histology, such as LG-ESS or adenosarcoma [7, 10, 11]. In contrast, in early-stage cases of endometrial carcinoma and cervical cancer, the risk of recurrence is defined by surgical pathological findings, and postoperative adjuvant therapy is selected according to a flowchart [7, 15]. Therefore, if risk factors for recurrence are identified in cases of complete resection of early-stage uterine sarcoma, additional surgery and postoperative adjuvant therapy may be best performed according to the risk.

Although several reports have investigated poor prognostic factors, including the risk of uterine sarcoma recurrence, most have comprehensively investigated all stages of the disease, while only a few have focused on early-stage cases.

First, several reports have identified prognostic factors in early-stage ULMS based on hematological or pathological findings. In a study of prognostic factors of OS in 159 patients with stage I ULMS in Japan, univariate analysis showed that high serum LDH (> 191 IU/L), large tumor size (> 5 cm), and presence of necrosis were associated with poor prognosis, whereas multivariate analysis identified high serum LDH (> 191 IU/L) as factors significantly associated with poor prognosis [8]. This study has the largest number of cases in Japan at present. In a Norwegian study of prognostic factors in ULMS cases with tumors confined to the uterus, that is, stage I, prognosis was associated with tumor size (*p* < 0.0001) and mitotic index (MI) (*p* < 0.0001) in univariate analysis. In multivariate analysis, tumor size (using 100 mm as a cutoff) was a strong independent prognostic factor (*p* < 0.001) with a relative hazard (RH) of 2.7, while MI (using 10/10 HPF as a cutoff) also had an RH of 1.9 [3]. Tumor grade has been reported to be associated with prognosis in stage I ULMS [16, 17], while mitotic counts have also been reported to be associated with prognosis in stage I ULMS [18].

To the best of our knowledge, there have been no large reports that investigated prognostic factors completely restricted to early-stage LG-ESS on hematology or pathological findings. Although the description of “ESS” alone may include HG-ESS, in a Norwegian study of prognostic factors in ESS cases with tumors confined to the uterus, that is, stage I, in which prognosis was associated with MI (*p* = 0.0007), tumor cell necrosis (*p =* 0.002), and cellular atypia (*p* = 0.029) in univariate analysis. In multivariate analysis, MI (RH = 4.1) and tumor cell necrosis (RH = 3.5) were significant prognostic factors [3]. In a study of mostly early-stage LG-ESS cases, 67 of 68 cases were stage I or II, and the mitotic activity index (MAI) was a significant prognostic factor (0–3 vs. > 3, *p* = 0.005, HR 8.6) [19]. The prognostic factors in cases of early-stage ULMS or LG-ESS in this study were similar to those previously reported.

This study demonstrated that a high mitotic count is associated with increased recurrence in stage I ULMS after complete resection at primary surgery. Previous reports have shown variations in terms such as MI, MAI, and mitotic count [3, 18, 19]. However, the overall conclusion that “cases with a high number of mitoses have poorer prognosis” was consistent with this study’s findings. We discuss the mechanism of this result. A high mitotic count reflects a high proliferative capacity, leading to rapid tumor growth. This means even minimal residual disease or micrometastases can proliferate rapidly and potentially lead to recurrence [20, 21]. Additionally, tumors with rapid proliferation tend to be rich in angiogenesis, leading to a higher risk of hematogenous metastasis to the lung and liver [22, 23]. Furthermore, rapid cell proliferation increases genomic instability, which can lead to treatment resistance and high aggressiveness [24]. We conclude that high mitotic counts are associated with increased recurrence and poor prognosis through these mechanisms.

Furthermore, there have been some reports of prognostic factors in early-stage cases of uterine sarcoma regarding surgical methods. Intraperitoneal morcellation is a poor prognostic factor in early-stage ULMS, increasing intraperitoneal recurrence and significantly shortening disease- or recurrence-free survival and OS [14, 25, 26]. In LG-ESS, which is dependent on estrogen, ovarian preservation has been reported not increase mortality in stage I [27], but to increase the recurrence rate [12, 13]. The tumor recurrence rate was 24.2% in the BSO group compared to 46.8% in the ovarian preservation group (OR: 2.70) [12], and the PFS for patients who underwent BSO vs. ovarian preservation was 38 vs. 11 months (*p* = 0.071) [13]. However, these reports partially included stage III or IV. On the other hand, numerous reports indicate that ovarian preservation does not affect prognosis in early-stage ULMS [16, 28]. Conversely, a report indicates that ovarian preservation cases tend to have a better prognosis than BSO cases in stage I ULMS [27]. However, approximately 71% of ULMS cases are reported to express ER [29], and ovarian preservation may be a poor prognostic factor in this population. The results of this study also suggested that ovarian preservation may be associated with recurrence in stage I ULMS, but we lacked sufficient information on ER/PR status to conduct a detailed analysis. The NCCN guidelines state that intraperitoneal morcellation is contraindicated in uterine sarcoma and that BSO is recommended to reduce the risk of recurrence in LG-ESS or tumors expressing ER/PR. However, none of the previously reported prognostic factors have been clearly identified in the NCCN guidelines as risk factors that contribute to treatment choice.

This study has some limitations. First, because this study was conducted at a single institution and the number of cases was small, only one factor showed a statistically significant difference. In addition, only univariate analysis could be performed; multivariate analysis could not. Thus, the limited sample size in this study may reduce statistical power and could affect the validity of the results. Further studies, in many cases across multiple institutions, will be necessary in the future. Second, because of the characteristics of a university hospital, patients who are difficult to treat or who have recurred are often referred, which may result in a shorter OS and PFS than expected. Third, for example, MIB-1 positivity rate and ER/PR expression were not measured in all cases in this study due to a retrospective study design involving multiple pathologists handling cases over long periods, the availability of immunohistochemical tests, and other factors. This point could further fulfill the evaluation of the pathological finding. New findings may be obtained by requesting pathologists to evaluate these factors in all cases going forward.

## Conclusions

In conclusion, it is suggested that some of the primary surgical methods and pathological findings may be associated with an increased risk of recurrence after complete resection of stage I ULMS and LG-ESS. A high mitotic count was significantly associated with recurrence in stage I ULMS. A multicenter collaborative study is currently underway to investigate more cases, with the aim of contributing to the establishment of evidence in Japan for the future.

## Data Availability

The datasets analyzed during the current study are available from the corresponding author on reasonable request.

## Abbreviations

BSO: Bilateral salpingo-oophorectomy
ER: Estrogen receptor
HG-ESS: High-grade endometrial stromal sarcoma
LG-ESS: Low-grade endometrial stromal sarcoma
LVSI: Lymphovascular space invasion
MAI: Mitotic activity index
MI: Mitotic index
NCCN: National Comprehensive Cancer Network
OS: Overall survival
PFS: Progression-free survival
RH: Relative hazard
STUMP: Smooth muscle tumor of uncertain malignant potential
ULMS: Uterine leiomyosarcoma
PR: Progesterone receptor
